# The Point of Death

**Published:** 1886-11

**Authors:** 


THE POINT OF DEATH.—*When Dissolution Occurs a Matter of Grave Uncertainty.*—Although usually it is easy to tell when dissolution has occurred, yet there are cases which occur from time to time rendering the matter one of very grave uncertainty. The point at which the soul relinquishes the body is among the most difficult things to establish. Perhaps this uncertainty is one of the reasons why there is such curiosity as to death-beds and last words. We are anxious to know how affairs appear to whose who are passing away. They are undergoing the great change through which every one must pass.

How does it look to them? Very little more information is to be gathered from the dying than from the dead. Certain inferences may be drawn from the surroundings—the departing color, the cold, deepening stare, the groan, the rattle in the throat, the stiffening limbs: but they are as likely to mislead as not. And the same may be said of the death sayings. They are as enigmatic as the declarations of the oracles. We may take sometimes half a dozen meanings from them, as, for instance, Goethe's "More light!" Was it the sense of earthly darkness growing around him, or was it the breaking of the eternal light upon his vision. or was there yet some deeper significance in the exclamation?

Medical works show that people have been resuscitated fifteen, twenty, and even thirty minutes after apparent death. Heldon, the highwayman, is said to have been dead three-quarters of an hour. His body was cut down after hanging that length of time, and was handed over to his friends after a thorough examination. That night he was seen as well as ever except for a stiffness of the neck. Pryce, the Norwich miser, was dead as Cæsar, according to those about him. and until some thoughtful person, distrusting the warmth of his hands, administered a stimulant. He arose and lived years afterward. Cases of mere trance are almost innumerable.

Supposed deaths from drowning show that resuscitation may take place thirty or forty minutes after all life has apparently left the body. The question as to what becomes of the soul in this long interval is the one which puzzles many. But the chief point of the matter is that the physicians and friends should not too readily accept appearances in the critical hour. There may, as in the Frazer instance, be yet some spark of life remaining. As Dr. Lackerstein claims, there is absolutely no reason why, with the resources he employed at hand, any one should die of an overdose of chloroform or from a shock while undergoing a surgical operation.—*Ex.*

